# Innovative Quality-Assurance Strategies for Tuberculosis Surveillance in the United States

**DOI:** 10.1155/2012/481230

**Published:** 2012-05-17

**Authors:** Lilia Ponce Manangan, Cheryl Tryon, Elvin Magee, Roque Miramontes

**Affiliations:** Division of Tuberculosis Elimination, National Center for HIV/AIDS, Viral Hepatitis, STD, and Tuberculosis Prevention, Centers for Disease Control and Prevention (CDC), Mailstop E-10, 1600 Clifton Road, Atlanta, GA 30033, USA

## Abstract

*Introduction*. The Centers for Disease Control and Prevention (CDC)'s National Tuberculosis Surveillance System (NTSS) is the national repository of tuberculosis (TB) data in the United States. Jurisdictions report to NTSS through the Report of Verified Case of Tuberculosis (RVCT) form that transitioned to a web-based system in 2009. *Materials and Methods*. To improve RVCT data quality, CDC conducted a quality assurance (QA) needs assessment to develop QA strategies. These include QA components (case detection, data accuracy, completeness, timeliness, data security, and confidentiality); sample tools such as National TB Indicators Project (NTIP) to identify TB case reporting discrepancies; comprehensive training course; resource guide and toolkit. *Results and Discussion*. During July–September 2011, 73 staff from 34 (57%) of 60 reporting jurisdictions participated in QA training. Participants stated usefulness of sharing jurisdictions' QA methods; 66 (93%) wrote that the QA tools will be effective for their activities. Several jurisdictions reported implementation of QA tools pertinent to their programs. Data showed >8% increase in NTSS and NTIP enrollment through Secure Access Management Services, which monitors system usage, from August 2011–February 2012. *Conclusions*. Despite challenges imposed by web-based surveillance systems, QA strategies can be developed with innovation and collaboration. These strategies can also be used by other disease programs to ensure high data quality.

## 1. Introduction

In 2010, there were 8.8 million new cases of tuberculosis (TB) disease reported worldwide, with over 1 million TB deaths [1]. In the United States, 11,182 people were newly diagnosed with TB disease [2]. The mission of the Division of Tuberculosis Elimination (DTBE), Centers for Disease Control and Prevention (CDC) is to promote health and quality of life by preventing, controlling, and eventually eliminating TB from the United States, and by collaborating with other countries and international partners in controlling TB globally [3]. 

Tuberculosis surveillance is a core public health function. Ongoing and systematic collection, analysis, interpretation, and dissemination of surveillance data allow programs to target control interventions that provide the most impact in eliminating TB [4]. These surveillance data are essential in describing morbidity and mortality, monitoring trends in TB incidence and prevalence, detecting potential outbreaks, and defining high-risk groups. In addition, TB data are needed to evaluate TB control programs, identify deficiencies, and allocate resources. In order to perform these important functions, it is essential that surveillance data are collected and reported in an accurate, complete, and timely manner.

The CDC's National Tuberculosis Surveillance System (NTSS) is the national repository of TB surveillance data in the United States. CDC receives data on TB cases from reporting jurisdictions through a standardized data collection form, the Report of Verified Case of Tuberculosis (RVCT). NTSS has 60 reporting jurisdictions: all 50 US states, the District of Columbia, New York City, American Samoa, Federated States of Micronesia, Guam, Republic of the Marshall Islands, Commonwealth of the Northern Mariana Islands, Puerto Rico, Republic of Palau, and US Virgin Islands.

The RVCT was revised by a group of TB experts in 2009 and transitioned into a new web-based reporting system. An interdisciplinary CDC DTBE team collaborated with key national partners, state-based medical or health officers, and other local healthcare professionals to launch a national training program on the new RVCT [5, 6]. Extensive reviews of training materials enabled partners to provide feedback for improvements on the instructions for each of the 49 RVCT items [7]. The team also developed a self-study manual for participants that was used during facilitator-led trainings [8]. The manual can also be used as self-study for new TB staff and as a reference guide. In addition, a facilitator manual was developed and used during training-of-trainers courses to build RVCT training capacity throughout the reporting jurisdictions [9].

Quality assurance (QA) is a critical part of any successful surveillance system and is a continuous cycle of monitoring, evaluating, and improving data quality [10, 11]. Prior to 2009, jurisdictions depended on a CDC disk operating system (DOS) used for surveillance of TB data. This system provided a series of validation reports to jurisdictions for managing data. When CDC transitioned to a web-based system in 2009, there was a need for a standardized QA process that jurisdictions could adapt to their setting.

The team determined that a logical followup to the RVCT trainings was to enhance the QA knowledge and skills of TB surveillance staff. Furthermore, the RVCT training participants expressed concerns regarding the lack of data validation of some state systems and the inability of reporting areas to transmit all data electronically. DTBE staff began working individually with state public health partners to develop QA strategies. This paper describes these strategies to ensure the quality of TB data reported to the CDC's NTSS through the new web-based system.

## 2. Materials and Methods

The RVCT QA training team, in collaboration with key partners, developed innovative strategies to provide standardized methodologies, skills, and tools to enhance the capacity for conducting QA. Similar to the RVCT training course, the team used the systematic process for health education to develop these QA strategies [6, 12]. This process includes needs assessment, development, pilot testing, implementation, and outcome evaluation.

### 2.1. Quality Assurance Needs Assessment

During 2010-2011, the training team conducted a comprehensive needs assessment to determine strategies that could enhance QA for TB surveillance data. During the needs assessment, the team facilitated discussions of QA topics with prepared open-ended questions to jurisdictions and CDC staff.

The needs assessment included the following.

- meeting with TB program area staff from 11 reporting jurisdictions in either focus groups or individual interviews. Three incidence levels of TB-burden areas were represented including low (≤3.5 cases per 100,000 population in 2009), medium (3.6–3.8 cases per 100,000 population in 2009), and high TB incidence (>3.8 cases per 100,000 population in 2009). The staff described their surveillance system, and staff characteristics (training and expertise level), and shared their QA process and tools (i.e., tables, charts, graphs, processes, and templates). Staff suggested content topics and prioritized QA components that should be covered in the materials and a training course. In addition, they discussed successes and challenges experienced when conducting QA at their sites;
- meeting with colleagues from DTBE who have a role in ensuring quality data including the subject matter experts in the laboratory, the Data Management and Statistics Branch, project officers for the National TB Indicators Project (NTIP), and the TB Genotyping Information Management System [13, 14]. These staff members collaborated to help develop and conduct a comprehensive training program and QA tools;
- meeting with surveillance staff from other CDC divisions including the Division of STD Prevention, Division of Viral Hepatitis, and the Division of HIV/AIDS Prevention. Some of these colleagues indicated they conducted QA only after data arrived at CDC. None of the QA procedures or processes utilized by other divisions met the needs of DTBE;
- conducting a review of available QA materials on surveillance data [15–38]. This review yielded information on various QA components and definitions (Table 1). However, the team did not find a comprehensive QA framework, practical step-by-step QA strategies for TB surveillance data, or practical models for a QA training course;
- reviewing the surveillance section of the Tuberculosis Elimination and Laboratory Cooperative Agreement, a portion of an agreement between DTBE and NTSS reporting jurisdictions that describes area surveillance activities [39]. This yielded QA components and a requirement to monitor data quality (Table 1).

### 2.2. Quality Assurance Strategies

The results of the needs assessment were used to develop 4 strategies for enhancing QA procedures in reporting jurisdictions. These include the following.

**(1) Providing a QA Process That Includes Five Components and Categorizing Activities into Each of These Components**

QA components include case detection, data accuracy, data completeness, data timeliness, and data security and confidentiality. These components provided logical steps for conducting QA activities and were designed to allow reporting areas to utilize those strategies that would benefit them.

**(2) Providing QA Tools Including Guidance for a Written QA Protocol**

The RVCT QA training team provided a template for a written QA protocol and other tools that jurisdictions can easily adapt and use to conduct QA. Staff from CDC and the various jurisdictions developed over 45 tools that were classified into each of the five QA components (Table 2). The tools include tables, charts, graphs, processes, and templates and are available in common electronic formats (e.g., Word, Excel, and PowerPoint). The team developed a main tool which is a template to help jurisdictions write a QA protocol required in the annual DTBE Cooperative Agreement (Table 2).

The National Tuberculosis Indicators Project (NTIP) is also an important QA tool [13]. During 2010, an NTIP module was developed to allow users to identify any TB case reporting discrepancies. This module has proven useful in recognizing data coding errors and data transmission problems, and highlighting the issue that errors are occurring more frequently than was previously recognized.

The reporting jurisdictions can access their NTIP and NTSS QA reports such as the missing and unknown (MUNK) reports through the Secure Access Management Services (SAMS). SAMS is a federal information technology system that gives authorized personnel secure, external access to nonpublic CDC applications.

**(3) Developing and Conducting a QA Training Course**

The training team developed and conducted a comprehensive QA training course to enhance the knowledge and skills needed by TB surveillance staff from the reporting jurisdictions for conducting QA. The results of the needs assessment and collaboration with subject matter experts were key to development of the QA course. The course focused on the QA process and five components, as well as other related topics to increase course participants' use of NTSS data for QA and program planning.

The course format included presentations from faculty (DTBE subject matter experts) with slides and handouts, exercises to apply the content to realistic situations, interactive discussions to share experiences and answer questions, and tools to use or adapt to their setting. Participants also described how they conducted QA at their sites and provided examples of QA challenges they encounter.

**(4) Developing a Resource Guide and Toolkit**

The training team is currently developing a resource guide and toolkit that can be used as a QA reference guide or a training manual. It will include many of the materials developed for the course such as handouts, exercises to apply the content, glossary, and examples of the tools (Table 2). A companion CD will provide the tools in easy-to-use formats (Word, Excel, and PowerPoint) so that jurisdictions can adapt them to their own setting. The guide and toolkit will be available in a print-based format with a companion CD that includes the tools. In addition, the materials will be downloadable from the CDC website.

## 3. Results and Discussion

### 3.1. Quality Assurance Training Evaluations

In July 2011, the team facilitated a pilot test of the 2-day QA training course with eleven TB surveillance experts from various state and local TB programs. The participants provided suggestions on how to improve the materials, the presentations, and the course schedule. The comprehensive course evaluation included written evaluations with qualitative and quantitative questions; discussions at the end of each of the five QA components; an end-of-course written evaluation; observations by course faculty. The team revised the materials and training course based on the analysis of the evaluation results (Table 3).

The team and other faculty members also conducted four 2-day trainings in Atlanta, GA, between August and September 2011. Course participants included 61 TB surveillance staff. Participants from the four trainings completed an end-of-course evaluation form consisting of qualitative and quantitative questions.

Results of the combined responses from the pilot course and four trainings evaluations (Table 3) indicated that participants learned about the QA process and benefited from sharing information on how other jurisdictions implement the QA components at their sites. The 73 participants (from the pilot course and four trainings) represented 34 (57%) of the 60 NTSS reporting jurisdictions. The 34 jurisdictions represent more than 80% of all TB cases reported to CDC each year.

Of the 73 participants, 66 (93%) stated that the QA tools will be effective in helping them conduct QA in their programs (Table 3). Participants stated that some of the most important things they learned were the five QA components and how they relate to the requirements in the cooperative agreement for a written QA TB surveillance protocol. Most of the participants appreciated the assessment of programmatic needs and the effort that went into implementing the course.

### 3.2. Quality Assurance Strategies with Limited Resources

In developing innovative strategies for QA of TB surveillance data, a key question for all programs is how best to maximize the use of limited resources to ensure data quality. The design and flexibility of the guide and toolkit enable health care staff to learn about the QA process in a self-study format or as part of a facilitator-led training course. Also, providing the materials in print-based format and the internet ensures accessibility to the materials without additional resources. Gaining knowledge and skills to conduct QA helps reporting jurisdictions remain vigilant in maintaining high quality of surveillance data despite limited resources.

### 3.3. Impact of Quality Assurance Strategies

This QA project represents a significant improvement to NTSS because it compiles for the first time guidelines, step-by-step process, and tools for monitoring and improving the quality of TB surveillance data. Additionally, CDC noticed an unprecedented timeliness and accuracy of all the required RVCT variables needed for publication on TB surveillance data in the Morbidity and Mortality Weekly Report issue for the World TB Day on March 24, 2012. The process to obtain data has been much easier than previous years because of improved understanding between CDC and jurisdictions fostered at the QA training.

Although the team conducted training on the QA strategies less than a year ago, CDC staff have also noticed better collaboration among NTSS reporting jurisdictions in sharing QA tools. Several jurisdictions sent letters to CDC shortly after the training stating that they have implemented QA tools that were pertinent to their programs.

In addition, SAMS portal reports indicated a 10% increase of NTSS enrollment from August 2011 to February 2012 and an 8% increase of NTIP enrollment for the same period. But it may be premature to attribute these results to the QA strategies.

The team also attempted to obtain MUNK reports of the jurisdictions represented during the QA training to examine any QA improvement, but this information was not readily available. This information can compare RVCT data and how missing or validation issues occur from the previous year. However, this may not be a reliable measurement of the impact of the QA strategies because the MUNK reports are influenced by changes in the number of TB cases, complexity of data-related issues, changes to state-based systems, or staff turnover.

The impact of the QA strategies to the quality of TB surveillance data can be better evaluated after the jurisdictions have fully implemented them. A survey of their QA practices may systematically evaluate the importance of these strategies.

Despite the limitations, these QA strategies support TB policies, laws, and regulations as they equip local jurisdictions with a systematic set of processes and tools that may be used to fulfill the requirements of the cooperative agreement to monitor data quality. Also, since reporting of a patient with TB disease to health authorities is mandated by state laws, the guidance on case detection, data accuracy, completeness, and timeliness helps reporting areas in complying with these laws.

In addition, these strategies are essential in collecting accurate and reliable TB surveillance data that are critical to making decisions to meet DTBE's priorities: interrupt transmission of *Mycobacterium tuberculosis, *reduce TB in foreign-born populations, reduce TB in racial/ethnic minority populations, mitigate/reduce impact of multidrug-resistant and extensively drug-resistant TB, and reduce HIV-associated TB. Being vigilant in performing QA ensures high-quality data and ultimately helps accelerate progress toward elimination of TB in the United States.

## 4. Conclusions

Despite challenges imposed by various surveillance systems, economic constraints, and new diagnostic technologies, strategies for conducting QA can be developed with innovation and collaboration. Mobilizing the TB community to ensure high-quality data involves commitment, time, and energy of TB leaders and partners.

Guidelines, a step-by-step process, and tools for monitoring and improving the quality of TB surveillance data are essential for the TB community to effectively control TB. Future evaluation on the impact of these QA strategies will further demonstrate their importance in maintaining data quality.

## Figures and Tables

**Table:** (a) Case detection: Case detection is the discovery of the existence of a single instance of a specific disease or exposure, for example, tuberculosis (TB). This is a front-line surveillance activity and typically accomplished as a by-product of routine medical or veterinary care, laboratory work, or via an astute observer. The primary purpose is to find all patients with TB diagnosis to treat and prevent TB transmission and are reported to the TB surveillance system

Activities	Description	Data sources
Maintain a registry of TB cases.	Contains at a minimum the elements to produce data for the national TB case report, the revised Report of Verified Case of Tuberculosis (RVCT). All local jurisdictions should also have at least a log, if not a registry, that contains key demographic and clinical information on each reported TB suspect. Data on TB cases receiving diagnostic, treatment, or contact investigation services in the local jurisdiction, although not included in the annual morbidity total, should be included in the TB registry.	(i) TB suspect registries from all local jurisdictions.

Establish liaisons with appropriate reporting sources to enhance quality assurance (QA) of TB surveillance data.	Enhance identification, reporting, and followup of TB cases and suspects by establishing liaisons with appropriate reporting sources. Jurisdictions should provide a plan for case finding and how they will or have established appropriate liaisons. Thereafter, TB programs should provide periodic feedback and at minimum, an annual written report summarizing surveillance data to reporting sources.	(i) Hospitals. (ii) Clinics (e.g., TB and HIV/AIDS clinics). (iii) Laboratories performing tests for mycobacteria. (iv) Selected physicians (e.g., pulmonary and infectious disease subspecialists). (v) Correctional facilities. (vi) Community and migrant health centers. (vii) Pharmacies. (viii) Other public and private facilities providing care to populations with or at risk for TB.

Develop and implement active case detection activities.	At a minimum, ongoing active laboratory surveillance should be conducted by on-site visits in all areas to ensure complete reporting of all TB cases and suspects with positive acid-fast bacilli (AFB) smears and cultures for *M. tuberculosis*.	(i) Laboratory reports.

Evaluate the completeness of reporting of TB cases to the surveillance system.	Periodically (e.g., at least every two years) evaluate the completeness of reporting of TB cases to the surveillance system by identifying and investigating at least one population-based secondary data source to find potentially unreported TB cases.	Secondary data source, for example (i) statewide laboratory record review, (ii) pharmacy review, (iii) hospital discharge data review,
	Potential TB cases identified during the evaluation must be verified.	(i) medical records, (ii) physician, interviews, (iii) patient interviews.
	Reasons for nonreporting of TB cases should be determined and a plan for improvement developed and implemented.	

**Table:** (b) Data accuracy: Data accuracy means that the data recorded match exactly what happens in a clinical encounter, whether or not it is clinically appropriate. The primary purpose is to identify and correct errors in the surveillance data

Activities	Description	Data sources
Evaluate accuracy/validity of RVCT data.	At least annually evaluate the accuracy/validity of RVCT data by comparing RVCT data and the jurisdiction's TB registry data to original data sources.	(i) RVCT data collection form. (ii) Patients' medical records. (iii) TB registry database.

Assess knowledge, skills, and abilities of staff and provide training if needed.	Assess the knowledge, skills, and abilities of all existing personnel and new hires whose duties involve the collection and reporting of registry and RVCT data.	(i) Personnel files. (ii) Staff interviews. (iii) Observations and evaluations of staff skills.
	Provide training and evaluation. Training will focus on accurate and timely completion of the revised RVCT. All existing staff will be trained on the revised RVCT data collection, and new staff should be trained within 2 months of hire date.	

**Table:** (c) Data completeness: Data completeness means that the information submitted contains the mandatory set of data items. The primary purpose is to capture all the relevant data on TB patients on the RVCT to support and improve the function of the TB surveillance system

Activities	Description	Data sources
Maintain completeness for all RVCT variables.	TB case data will be reported to CDC using the revised RVCT form via an electronic format that conforms to Public Health Information Network (PHIN) and/or National Electronic Disease Surveillance System (NEDSS) messaging standards.	(i) RVCT form via an electronic format.
	HIV status will be reported for at least 95 percent of all newly reported TB cases, age 25–44 years.	(i) HIV reports.
	A valid genotype accession number (generated by the CDC-sponsored genotyping laboratory) will be reported for at least 85 percent of all reported culture-positive cases.	(i) Genotyping reports.
	TB programs will maintain at least 95 percent reporting completeness for all variables existing on the pre-2009 RVCT.	(i) Pre-2009 RVCT form.
	By 2013, TB programs will achieve 95% completeness of all variables in the revised RVCT.	(i) Post-2009 RVCT form.

Match TB and AIDS registries.	Collaborate with the HIV/AIDS program to conduct at least annual TB and AIDS registry matches to ensure completeness of reporting of HIV and TB coinfected patients to both surveillance systems. Investigate and verify all TB cases reported to the HIV/AIDS program and not reported to the TB program. Update the TB registry and report to CDC as needed. At least annually assess reasons for incomplete HIV results on the RVCT for each verified case of TB.	(i) TB registries. (ii) HIV/AIDS registries.
	Determine if patients were not tested for HIV or were tested but results not reported to the TB program. Develop and implement plans for improvement in increasing HIV testing and reporting to patients and TB programs.	

**Table:** (d) Data timeliness: Data timeliness is the speed between steps in the surveillance system. Data are current and available on time. The primary purpose is to ensure that data are available for TB program planning and for appropriate distribution of resources

Activities	Description	Data source
Report all newly diagnosed cases of TB to the CDC according to schedule.	Report all newly diagnosed cases of TB to the CDC according to a schedule agreed upon each year, generally monthly, and at least quarterly.	(i) RVCT reports.

Submit complete RVCT reports according to schedule.	The initial case reports should be submitted generally monthly and at least quarterly.	(i) RVCT report. (ii) Initial case report.
	Followup 1 report, which is only for TB cases with positive culture results, should be completed and submitted within 2 months after the initial RVCT was submitted, or when drug susceptibility results are available, whichever is later.	(i) RVCT reports. (ii) Followup 1 (Initial Drug Susceptibility Report).
	The followup 2 report, which should be submitted for all cases in which the patient was alive at diagnosis, should have data entered as it becomes available, and it should be complete when the case is closed to supervision. All followup 2 reports should be completed within two years of initial case reporting.	(i) RVCT reports. (ii) Followup 2 (Case Completion Report).

Analyze TB surveillance data at least quarterly.	At least quarterly, analyze TB surveillance data to monitor trends, detect potential outbreaks, and define high-risk groups. Produce and disseminate at least an annual report summarizing current data and trends.	(i) Surveillance data base.

Evaluate programmatic performance by using TB surveillance data at least annually.	At least annually, evaluate programmatic performance by using TB surveillance data to assist in compiling supporting evidence to determine the extent to which program objectives are being met and also to assist in developing strategies for improvement.	(i) National TB Indicators Project reports.

**Table:** (e) Data security and confidentiality: Data security is the protection of public health data and information systems against unauthorized access. Data confidentiality is the protection of personal information collected by public health organizations. The primary purpose of security is to prevent unauthorized release of identifying information and accidental data loss or damage to the systems, while confidentiality is to ensure that personal information is not released without the consent of the person involved, except as necessary to protect public health

Activities	Description	Data sources
Ensure that TB surveillance data are kept confidentially and that all data files are secure.	Policies and procedures must be in place to protect the confidentiality of all surveillance case reports and files.	(i) Data security and confidentiality policies and procedures of the TB program. (ii) Surveillance case reports and files.
	Policies and procedures to protect HIV test results must conform to the confidentiality requirements of the state and local HIV/AIDS programs.	(i) Confidentiality requirements of the state and local HIV/AIDS programs. (ii) Observation of staff.
	Provide training on security and confidentiality of data.	

*Adopted from the 2011 cooperative agreement between the Division of Tuberculosis Elimination, Centers for Disease Control and Prevention and all 60 reporting jurisdictions of the National Tuberculosis Surveillance System.

**Table:** (a) Quality assurance protocol tools

Tool number	Tool name	Description and how to use	Format
QA protocol-1	Cooperative agreement	The original version of the TB surveillance section of the cooperative agreement.	PDF

QA protocol-2	QA written protocol-requirements	A table that lists all of the cooperative agreement requirements for TB surveillance and sources for information.	Word

QA protocol-3	QA written protocol-guide	A guide to help jurisdictions write their own protocol.	Word

**Table:** (b) Case detection tools

Tool number	Tool name	Description and how to use	Format
Case detection-1	TB program area module (TB PAM) flow chart	Flow chart to help with patient search. This flow chart was created initially to emphasize the importance of always searching for a patient within TB PAM so that duplicate patient records are not created. This flow chart also outlines the process for creating “Provider Verified” cases and addresses approval and rejection of notification.	Word

Case detection-2	Notification process	Flow chart that shows a tiered notification process for TB case notification. This flow chart identifies (1) what role each person (with a particular TB PAM right) has in the notification process and what happens when a notification is rejected or approved. Only TB program managers (a nurse within the TB program) creates a notification, and it must be approved by the TB Program Central Office Epidemiologist before it is sent to CDC for case counting.	Word

Case detection-3	TB suspects weekly report	This report is generated weekly for all suspects reported in TB PAM through Friday of the previous week. Suspects are classified as a case or not a case within 56 days from the date of report. There is a built-in calculation that calculates 56 days from the date of report (when the date of report is entered). All suspects that are past due (over 56 days) require a followup from one of the Central Office Nurse Consultants.	Excel

Case detection-4	Case verification and treatment status	Table that indicates case verification and treatment status. This spreadsheet is used to monitor treatment progress with the goal of completing treatment within 12 months. There are built-in calculations for 3, 6, 9, and 12 months from treatment start that are populated when the date therapy started is entered. Case verification is included to help identify how long treatment is anticipated for.	Excel

Case detection-5	Decline in reported tuberculosis cases survey	Sample survey to investigate decline in reported TB cases.	Word

Case detection-6	Counted tuberculosis case verification report	Form that indicates counted TB case verification.	PDF

Case detection-7	Investigation process for underreporting of TB	Table that provides a process for investigating underreporting of TB data.	Word

Case detection-8a	TB case closeout letter	Sample letter to accompany TB case close list (tool 8b) and TB case closeout form (tool 8c).	Word

Case detection-8b	TB case close list	List by jurisdiction indicating TB case closeout status.	Excel

Case detection-8c	TB case closeout form	Form for confirmation/signature of number of TB cases.	Word

**Table:** (c) Accuracy tools

Tool number	Tool name	Description and how to use	Format
Accuracy-1a	Data accuracy checklist	A checklist for reviewing RVCT data for accuracy	Word

Accuracy-1b	Data accuracy checklist CDC SAS code	SAS code corresponding to the data accuracy checklist, accuracy tool-1a, and is based on CDC RVCT variable names.	Word

Accuracy-1c	CDC TB surveillance RVCT data dictionary	Data dictionary for interpreting the CDC RVCT variable names used in data accuracy checklist CDC SAS code, accuracy tool-1b	Excel

Accuracy-2	Options for prioritizing medical chart reviews when resources are limited	Various options to help prioritize medical chart reviews when resources are limited.	Word

Accuracy-3	RVCT surveillance database audit form	Checklist for checking the accuracy of RVCT.	Word

Accuracy-4	Accuracy checklist for sputum culture conversion	Table to indicate number of days for culture conversion by jurisdiction. This applies to cases that are sputum culture positive only. There are built-in calculations that calculate the date, that is, 30 and 60 days from treatment start (once the date therapy started is entered). There is also a built-in calculation for the number of days that it took for sputum culture conversion. This helps identify those patients who did not meet the National Tuberculosis Indicators Project (NTIP) Objective of converting their sputum culture within 60 days of treatment initiation.	Excel

Accuracy-5	Nucleic acid amplification test (NAAT) comparisons	Comparison of NAAT tests.	Excel

Accuracy-6	RVCT calculated variables	RVCT calculated variables algorithm for calculating vercrit.	Word

Accuracy-7	2009 RVCT form with public health information network (PHIN) variable identification	2009 RVCT form with PHIN variable identification by RVCT question number to use as a reference for report codes.	PDF

**Table:** (d) Completeness tools

Tool number	Tool name	Description and how to use	Format
Completeness-1	Source list for locating RVCT data	Source document to locate information for each item on the RVCT.	Word

Completeness-2	Therapy status	Table to indicate therapy status by 12-month interval. This spreadsheet is used to monitor treatment progress with the goal of completing treatment within 12 months. There are built-in calculations for 3, 6, 9, and 12 months from treatment start that are populated when the date therapy started is entered. This tool targets the NTIP objective of treatment completion within 12 months.	Excel

Completeness-3	Culture and drug susceptibility status	Table to indicate culture and drug susceptibility status by jurisdiction. This report shows the susceptibility results for isoniazid, rifampin, pyrazinamide, and ethambutol. This shows those cases that are multidrug-resistant TB and also those who have an unknown or blank susceptibility report. This is for all culture-positive TB cases. This tool targets the NTIP objective of drug susceptibility reporting.	Excel

Completeness-4	TB Program area module (TB PAM)	Flow chart that shows the TB PAM process (initiation of RVCT through case closure). This flow chart was created for the TB PAM from initiating the RVCT to closing a case. This flow chart also identifies the responsible person(s) for the various steps.	Word

Completeness-5	Data abstraction instructions	Detailed procedures for RVCT quality control queries.	Word

Completeness-6a	RVCT variables used in NTIP	List of the RVCT variables used in the NTIP indicator calculation.	PDF

Completeness-6b	RVCT variables used in NTIP spreadsheet	Spreadsheet of the list of the RVCT variables used in the NTIP indicator calculation.	Excel

**Table:** (e) Timeliness tools

Tool number	Tool name	Description and how to use	Format
Timeliness-1a	Building blocks—schedule for entering RVCT data	Timeline table. This is a building-block diagram addressing what RVCT variables should be entered and identifies the time frame when those variables should be entered. This helps field staff know when information should be available and when the state central office expects it to be entered.	Word

Timeliness-1b	Schedule for entering RVCT data	Timeline table similar to timeliness tool 1a. It is in a table format rather than the graphic of the building blocks.	Word

Timeliness-2	Quarterly case summary	Document that summarizes timeliness measures and objectives for a predetermined set of TB patients. Predefined case outcome objectives are presented for that particular set of TB patients.	Excel

Timeliness-3	Timeliness data dictionary	Description of the data used to calculate timeliness measures for analysis. These measures are used to determine completion of state objectives.	Word

Timeliness-4	2010 Final verbal case counts and data submissions	Spreadsheet for case count.	Excel

Timeliness-5	Timeline for reporting TB data to CDC	Timeline for reporting TB cases and final TB data transmissions to CDC.	PDF

Timeliness-6	Typical weekly data availability chart	Typical weekly data availability by day of the week.	PDF

**Table:** (f) Data security and confidentiality tools

Tool number	Tool name	Description and how to use	Format
Data security and confidentiality-1	Standards for data security and confidentiality	List of minimum standards to facilitate data sharing and use of surveillance data for public health action.	Word

Data security and confidentiality-2	Initial assessment of TB program data security and confidentiality	Guide for the initial assessment of TB program's data security and confidentiality.	Word

Data security and confidentiality-3	Checklist for ongoing assessment of programs	Comprehensive checklist for assessing data security and confidentiality.	Word

Data security and confidentiality-4	Data security and QA checklist	A TB program's checklist for data security and QA.	Word

**Table:** (g) Other tools

Tool number	Tool name	Description and How to Use	Format
Other-1	QA protocol example	Four-phase process for entering RVCT data, to conduct quality control, and ensure timeliness in reporting.	Word

Other-2	QA in surveillance literature sources	References for quality assurance of surveillance data.	Word

Other-3	2009 trending guidance	Mapping the old RVCT data to the new RVCT data and diagrams to illustrate the following three RVCT items: (i) 16—site of disease, (ii) 22A—X-ray, (iii) 46—type of health care provider. This document provides a visual explanation of the transition between old and revised RVCT variables. Mapping shows the user exactly how the definitions of previous variables match up with the new ones.	Word

Other-4	Cohort review preparation	Timeline for planning and conducting a cohort review session. Includes preparation timeline and job responsibilities. Determines when participants need to be notified of scheduled events leading up to the cohort review session.	Word

Other-5	TB case/suspect QA review form	A checklist to use when reviewing TB cases/suspects.	Word

Other-6	TB review and QA schedule	Quality assurance schedule for various reviews of TB cases/suspects.	Word

**Table 3:** Combined evaluation results of the quality assurance training courses, Centers for Disease Control and Prevention, July–September 2011.

Evaluation question	Combined responses from the pilot course and four trainings		
	Reponses *N* = 73		Participants' comments
Overall, how confident are you that the target audience can learn about QA after having attended this course?	Very confident/confident	99% (72)	“Great job! Please continue this class. It was very informative and I learned a lot.” “Identified a number of good suggestions on how to improve my states level of accuracy with new tools provided by different topic speakers.”

How effective will the QA process (as described in this course) help you conduct QA in your job?	Very effective/effective	100% (73)	“I learned a great deal from this pilot. This course will be valuable to the states and will lead to great discussions and changes in the way QA is performed. This will therefore lead to great improvement in the quality of the data.”

How effective will the tools be in helping you conduct QA in your job?	Very effective/effective	93% (66)	“Very informative—lots of info I can actually use and apply to day-to-day activities (i.e., tools)”
